# Pathogenesis of intracranial aneurysm is mediated by proinflammatory cytokine TNFA and IFNG and through stochastic regulation of IL10 and TGFB1 by comorbid factors

**DOI:** 10.1186/s12974-015-0354-0

**Published:** 2015-07-22

**Authors:** Sanish Sathyan, Linda V. Koshy, Lekshmi Srinivas, H. V. Easwer, S. Premkumar, Suresh Nair, R. N. Bhattacharya, Jacob P. Alapatt, Moinak Banerjee

**Affiliations:** Human Molecular Genetics Laboratory, Rajiv Gandhi Centre for Biotechnology, Thiruvananthapuram, 695014 Kerala India; Department of Neurosurgery, Sree Chitra Tirunal Institute for Medical Science and Technology, Thiruvananthapuram, Kerala India; Department of Neurosurgery, Calicut Medical College, Calicut, Kerala India

**Keywords:** Inflammation, Cytokine, Aneurysm, SNP, *TNFA*, *IL10*, *TGFB1*, Meta-analysis

## Abstract

**Background:**

Intracranial aneurysm (IA) is often asymptomatic until the time of rupture resulting in subarachnoid hemorrhage (SAH).There is no precise biochemical or phenotype marker for diagnosis of aneurysm. Environmental risk factors that associate with IA can result in modifying the effect of inherited genetic factors and thereby increase the susceptibility to SAH. In addition subsequent to aneurismal rupture, the nature and quantum of inflammatory response might be critical for repair. Therefore, genetic liability to inflammatory response caused by polymorphisms in cytokine genes might be the common denominator for gene and environment in the development of aneurysm and complications associated with rupture.

**Methods:**

Functionally relevant polymorphisms in the pro- and anti-inflammatory cytokine genes *IL-1* complex (*IL1A*, *IL1B*, and *IL1RN*), *TNFA*, *IFNG*, *IL3*, *IL6*, *IL12B*, *IL1RN*, *TGFB1*, *IL4*, and *IL10]* were screened in radiologically confirmed 220 IA patients and 250 controls from genetically stratified Malayalam-speaking Dravidian ethnic population of south India. Subgroup analyses with genetic and environmental variables were also carried out.

**Results:**

Pro-inflammatory cytokines *TNFA* rs361525, *IFNG* rs2069718, and anti-inflammatory cytokine *IL10* rs1800871 and rs1800872 were found to be significantly associated with IA, independent of epidemiological factors. *TGFB1* rs1800469 polymorphism was observed to be associated with IA through co-modifying factors such as hypertension and gender. Functional prediction of all the associated SNPs of *TNFA*, *IL10*, and *TGFB1* indicates their potential role in transcriptional regulation. Meta-analysis further reiterates that *IL1* gene cluster and *IL6* were not associated with IA.

**Conclusions:**

The study suggests that chronic exposure to inflammatory response mediated by genetic variants in pro-inflammatory cytokines *TNFA* and *IFNG* could be a primary event, while stochastic regulation of *IL10* and *TGFB1* response mediated by comorbid factors such as hypertension may augment the pathogenesis of IA through vascular matrix degradation. The implication and interaction of these genetic variants under a specific environmental background will help us identify the resultant phenotypic variation in the pathogenesis of intracranial aneurysm. Identifying genetic risk factors for inflammation might also help in understanding and addressing the posttraumatic complications following the aneurismal rupture.

**Electronic supplementary material:**

The online version of this article (doi:10.1186/s12974-015-0354-0) contains supplementary material, which is available to authorized users.

## Introduction

Intracranial aneurysm (IA) is often asymptomatic until the time of rupture resulting in subarachnoid hemorrhage (SAH).There is no precise biochemical or phenotype marker for diagnosis of aneurysm. Rupture of cerebral aneurysm is the foremost cause for spontaneous subarachnoid hemorrhage (SAH). Intracranial aneurysm accounts for 85 % of subarachnoid hemorrhage (SAH), which contributes for 5–15 % of strokes [1] but occurs at a fairly young age and accounts for 25 % of stroke-related mortality [2]. In an earlier study, we have reported that hypertension and smoking are causal risk factors which might also modify the effect of genetic factors resulting in increased susceptibility to aSAH in the Indian population [3]. Inherited and acquired risk factors indicate influence on hemodynamic factors, defects in the vessel wall which may be in the muscular layer (tunica muscularis) of arteries, or alterations in the internal elastic membrane (lamina elastica interna) of cerebral arteries [4]. It is believed that hemodynamic stress can trigger localized inflammatory infiltration leading to formation of intracranial aneurysm by thinning and weakening in intracranial vessel [5]. Studies have pointed towards the presence of inflammatory cells such as macrophages, T and B lymphocytes in the wall of intracranial arteries [6].Various expression studies carried out till date with respect to intracranial aneurysm, support the role of inflammation and immune response with intracranial aneurysm [7]. These reports clearly demonstrate that inflammation plays a pivotal role in the pathogenesis of intracranial aneurysm.

Inflammation is an essential component of the immune response which involves inflammatory factors such as cytokines. When endothelial cells undergo inflammatory activation, it recruits cytokines, growth factors, and matrix metalloproteinase’s (MMPs). Balance between pro-inflammatory and anti-inflammatory cytokines defines the control of inflammation [8]. If vascular inflammation progresses unresolved, it can lead to vascular disease, whereas delayed inflammatory response can lead to ECM deposition, granular tissue formation and connective tissue growth. One would presume that genetic variability in cytokine genes can result in differential activation of immune response. The present study intends to explore the genetic role of pro-inflammatory and anti-inflammatory cytokine genes in intracranial aneurysm in a south Indian population. The study intends to evaluate the functionally relevant polymorphic variants of pro-inflammatory and anti-inflammatory cytokine genes [IL-1 complex (*IL1A, IL1B*, and *IL1RN*), *TNFA*, *IFNG*, *IL3*, *IL6*, *IL12B*, *IL1RN*, *TGFB1*, *IL4*, and *IL10]* and their role in aSAH. The role of these cytokine variants in influencing the epidemiological risk factors such as hypertension, gender and smoking and its relevance in global perspective will also be examined in the present study.

## Materials and methods

### Study population

The study populations consist of radiologically confirmed 220 IA cases and 250 ethnically and age-matched controls from the Malayalam-speaking Dravidian ethnic population of south India. The study was restricted to Malayalam-speaking Dravidian ethnic population of south India to avoid population stratification issues [9]. Patients having saccular intracranial aneurysm with aSAH were initially screened by CT and were angiographically confirmed by magnetic resonance angiography (MRA) and digital subtraction angiography (DSA). Exclusion criteria include patient with non-saccular aneurysm, arteriovenous malformation (AVMs), and other hereditary connective tissue disorders like autosomal dominant polycystic kidney disease, Marfan syndrome, and Ehlers-Danlos syndrome. Cases were recruited from two main tertiary care neurosurgical centers in Kerala. All the patients were rated based on WFNS scale. The control population consists of age-, sex-, and ethnicity-matched individuals who were symptomatically normal and did not possess any symptoms or family history of intracranial aneurysm. All the study subjects gave informed, written consent in a standard consent form to participate in the study after being provided with and receiving a full explanation of study protocols and objectives. The present study was approved by the institutional ethics committee of Rajiv Gandhi Center for Biotechnology, established as per the Indian council of medical research guidelines. Peripheral blood was collected from the study subjects in EDTA-coated vials, and DNA was isolated by conventional phenol-chloroform method.

### SNP selection and genotyping

SNPs were selected based on the functional relevance and minor allele frequency using genotype data obtained from Caucasian individuals in the HapMap project (HapMap Data Rel 24/Phase II Nov08, on NCBI B36 assembly, dbSNP b126). Some of the SNPs of *IL6*, *IL12B*, *IL1A*, and *IL1B* were selected based on their associations in the previous studies. Genotyping of 22 SNPs from (*IL1A*, *IL1B*, and *IL1RN*), *TNFA*, *IFNG*, *IL3*, *IL6*, *IL12B*, *IL1RN*, *TGFB1*, *IL4*, and *IL10* was carried out using TaqMan allelic discrimination assays (Applied Biosystems, Foster City, CA, USA) and KASPar method (KBioscience, Hoddesdon, UK) (Additional file 1: Table S1). TaqMan and KASPar reactions were performed in a 5-μl volume according to the manufacturer’s instructions on a 384-well-based ABI7900HT thermo cycler. Detection was performed using an ABI PRISM 7900 HT sequence detection system with SDS 2.4 software (Applied Biosystems, Foster City, CA, USA).

### Statistical analysis

Genotype and allelic frequencies were computed and were tested for deviation from Hardy-Weinberg equilibrium (ihg2.helmholtz-muenchen.de/cgi-bin/hw/hwa1.pl). All statistical analyses were performed using the GraphPad Prism 5.01, San Diego, CA, USA. We considered *p* value of <0.05 as significant. Chi-square test, allelic odds ratios (OR), and 95 % confidence intervals (CI) were calculated by Fisher’s exact test (two-tailed). To estimate linkage disequilibrium (LD) between pairs of loci in the patient and control populations, standardized disequilibrium coefficient (D′) and squared correlation coefficient (*r*^2^) were calculated using Haploview 4.1 (www.broad.mit.edu/mpg/haploview/). LD blocks were defined in accordance with Gabriel’s criteria. Further stratification of the patients was done to understand the role of associated cytokine gene variant between genders and hypertensive and non-hypertensive groups.

Functional prediction of the deleterious effect if any of the associated SNPs with respect to the functional categories such as protein coding, splicing regulation, transcriptional regulation, and post-translation was assessed in silico using F-SNP program (compbio.cs.queensu.ca/F-SNP/), FastSNP (fastsnp.ibms.sinica.edu.tw), SNPNexus (snpnexus.org), HaploReg (broadinstitute.org/mammals/haploreg), and regulomeDB (regulome.stanford.edu). F-SNP extracts information from large number of resources such as PolyPhen, SIFT, SNPeffect, SNPs3D, LS-SNP, Ensembl, ESEfinder, RescueESE, ESRSearch, PESX, TFSearch, Consite, GoldenPath, KinasePhos, OGPET, and Sulfinator to generate a functional significance (FS) score.

A meta-analysis with the random effects and fixed effects model was performed for previously studied SNPs using Review Manager 5.2 (reviewmanager.software. informer.com/5.2/). All the previous studies which explored similar variants in genes involved in inflammatory and anti-inflammatory cytokines were included in this study. The meta-analysis included the most studied SNPs in immune response, rs1800796 of *IL6* [10–14], rs3212227 of *IL12B* [15], and rs16944 of *IL1B* [16, 17] (Additional file 1: Table S2). The inconsistency index *I*^2^ was used to assess between-study heterogeneity. A *p* value of <0.05 was considered as significant throughout the analyses.

### Results

**Fig. 1:**
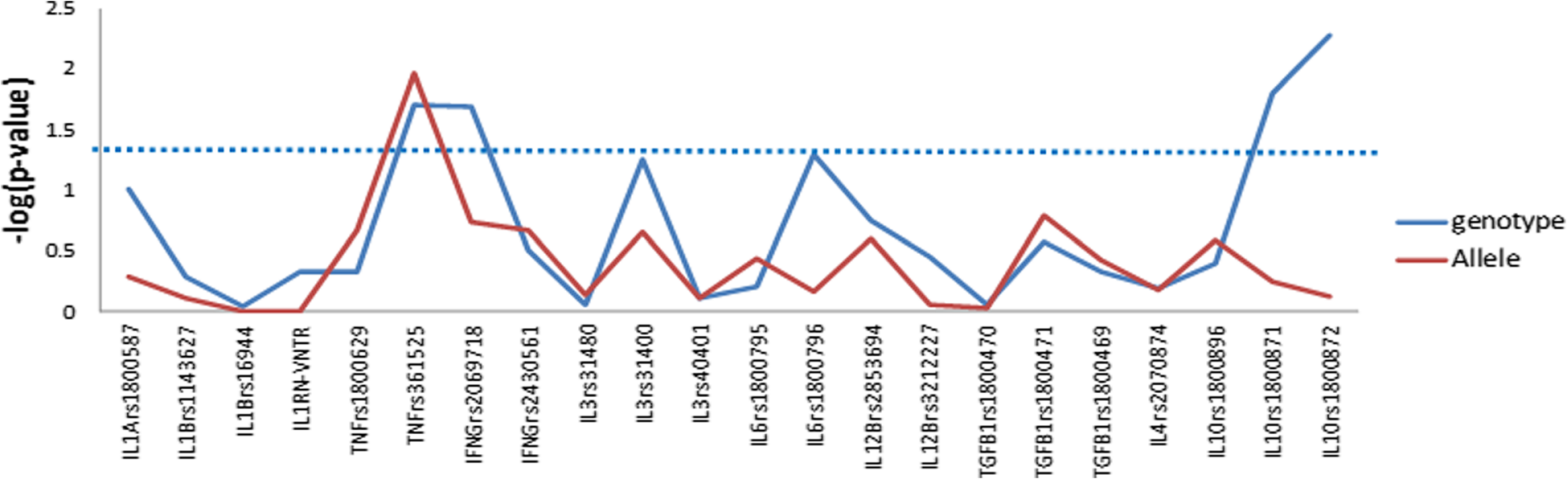
Genetic association analysis with studied variants between IA patients and controls. SNPs were plotted against –log(*p* value)

The demographic and clinical characteristics of subjects were recorded and are summarized in Table 1. All the studied variants were in Hardy-Weinberg equilibrium (*p* > 0.05) in healthy controls. Using a case control design, we observed a novel association with pro-inflammatory cytokines *TNFA* and *IFNG* (Table 2, Fig. 1). In *TNFA* rs361525 was found to be associated with IA at allelic (*p* = 0.019, OR 1.89, CI 1.16 to 3.06) and genotypic level (*p* = 0.010). In case of *IFNG*, of the two variants screened (rs2069718 and rs2430561), a genotypic association was observed for rs2069718 (*p* = 0.020). However, some of the extensively studied pro-inflammatory cytokine variants such as rs1800587of *IL1A* and rs1143627 and rs16944 of *IL1B* in *IL1* gene cluster were not found to be associated with IA in south Indian population (Additional file 1: Table S3). Similarly with *IL6* variants too, we could not observe a major significant association although *IL6* variants rs1800796 (−572C/G) indicated a trend towards association (Table 2) with IA in the present study population. The SNPs in pleiotropic cytokine genes *IL3*, *IL12B*, *IL4*, and anti-inflammatory gene *TGFB1* were also not found to be associated with intracranial aneurysm (Additional file 1: Table S3). However, two linked SNPs of anti-inflammatory cytokine *IL10* rs1800871 and rs1800872 indicated an association with heterozygous genotype with *p* value of 0.016 and 0.005, respectively, with IA (Table 2).The frequency of these variants vary widely across different world population. In silico functional prediction results for all the associated SNPs indicate that *TNFA* rs361525 and *IL10* rs1800871 and rs1800872 have potential role in transcriptional regulation (Table 2, Additional file 1: Table S4).

**Table 1:** Clinical characteristics of patients

Patient characteristics	
Mean age ± SD, years	51.17 ± 11.37
Men, %	55.7
Women, %	44.2
History of hypertension,%	35
History of diabetes, %	5.4
Family history of aSAH, %	3.7
Cigarette smoking, %	42.92
Alcohol use, %	21.8
Intracranial aneurysm location	Percentage
Anterior communicating artery	39.21
Anterior cerebral artery	8.37
Middle cerebral arteries	22.91
Internal carotid artery	17.18
Posterior communicating artery	8.81
Basilar artery	2.64
Posterior cerebral artery	0.44
Vertebral arteries	0.44

**Table 2:** Comparison of the genotype and allele frequencies of associated cytokine gene variants between patients and controls

					*p* value			OR(95 %CI)	*p* value	FS score
IFNG		AA	AG	GG		A	G			
rs2069718	Cases	79	103	39	0.020	261	181	1.20 (0.926 to 1.560)	0.184	ND
		0.36	0.47	0.18		0.59	0.41			
	Controls	61	143	39		265	221			
		0.25	0.59	0.16		0.55	0.45			
TNF		GG	AG	AA		G	A			
rs361525	Cases	171	42	2		384	46	1.89 (1.163 to 3.064)	0.010	0.208
		0.80	0.20	0.01	0.019	0.89	0.11			
	Controls	216	25	2		457	29			
		0.89	0.10	0.01		0.94	0.06			
IL6		GG	GC	CC		G	C			
rs1800796	Cases	57	126	37	0.050	240	200	0.94 (0.729 to 1.225)	0.691	0.208
		0.26	0.57	0.17		0.55	0.45			
	Controls	81	111	52		273	215			
		0.33	0.45	0.21		0.56	0.44			
IL10		CC	CT	TT		C	T			
rs1800871	Cases	48	142	40	0.016	238	222	1.08 (0.837 to 1.396)	0.558	0.101
		0.21	0.62	0.17		0.52	0.48			
	Controls	61	118	62		240	242			
		0.25	0.49	0.26		0.50	0.50			
IL10		AA	AC	CC		A	C			
rs1800872	Cases	39	143	48	0.005	221	239	0.955 (0.740 to 1.233)	0.745	0.101
		0.17	0.62	0.21		0.48	0.52			
	Controls	62	116	66		240	248			
		0.25	0.48	0.27		0.49	0.51			

*FS score* functional significance score, *ND* not determined

**Fig. 2:**
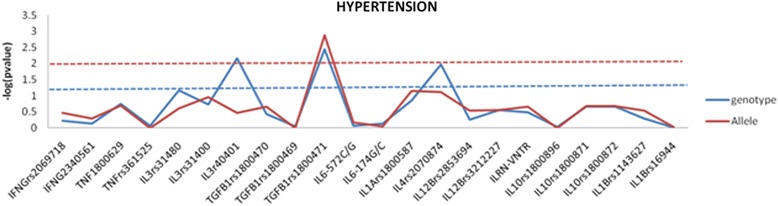
Genetic association analysis with studied variants between hypertensive and non-hypertensive IA. SNPs were plotted against –log(*p* value)

Stratification of data based on hypertension and gender was further carried out within the patient group to identify whether genetic association for IA was influenced by hypertension or gender or independent of these factors. We could not find any significant association for this stratification analysis with exception to *TGFB1* Arg25Pro rs1800471. The SNPs that were associated with IA were not influenced by genetic association of hypertension (Fig. 2) or gender (Additional file 1: Figure S1). Interestingly, in the subgroup analysis, *TGFB1* rs1800471 which was not associated with disease alone was found to be associated with hypertension-mediated resultant IA (Fig. 2).

**Fig. 3:**
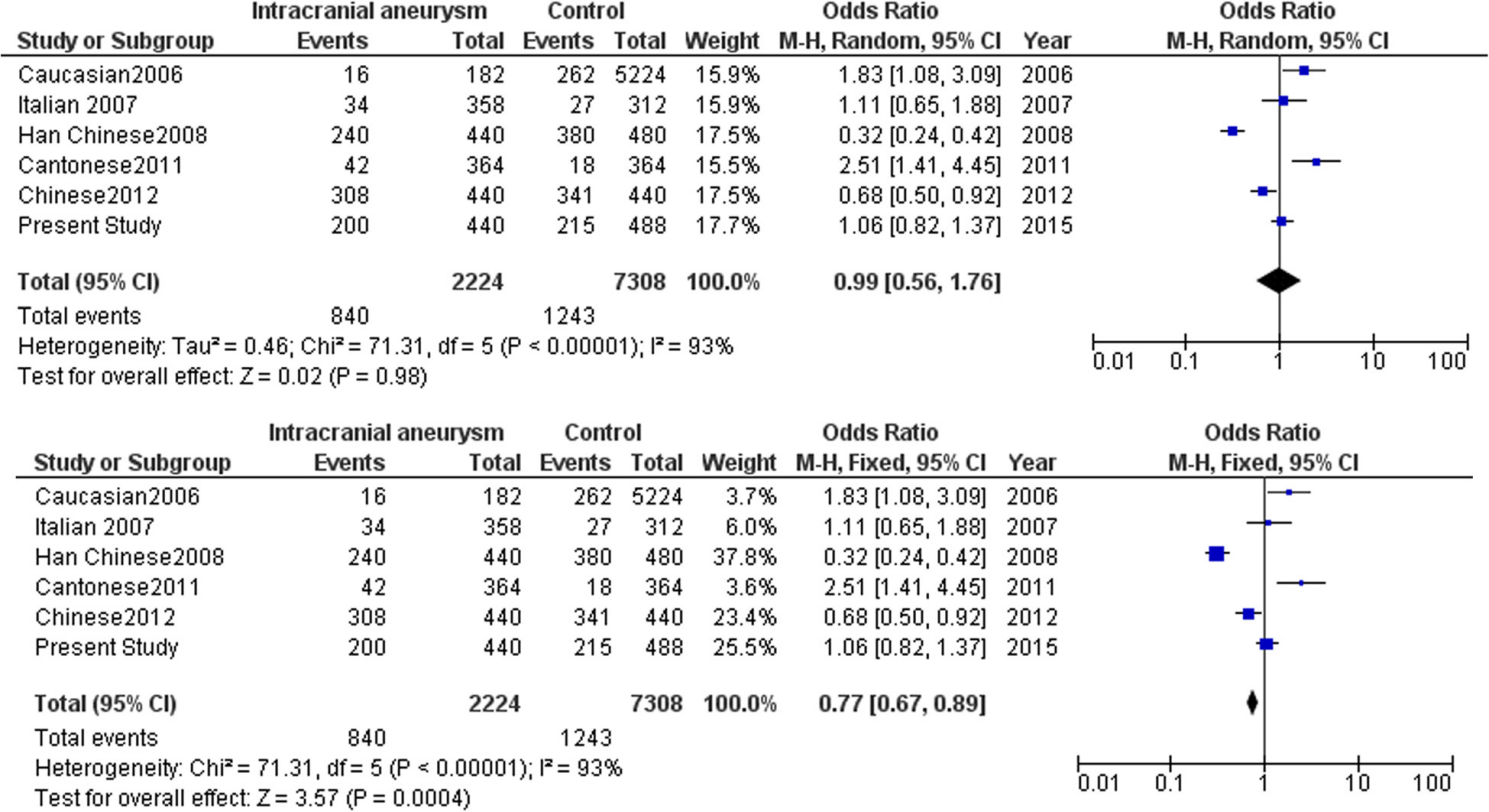
Meta-analysis of *IL6* rs1800796 (−572C/G) C versus G allele in patients with intracranial aneurysm versus normal control. Random and fixed effect models

**Fig. 4:**
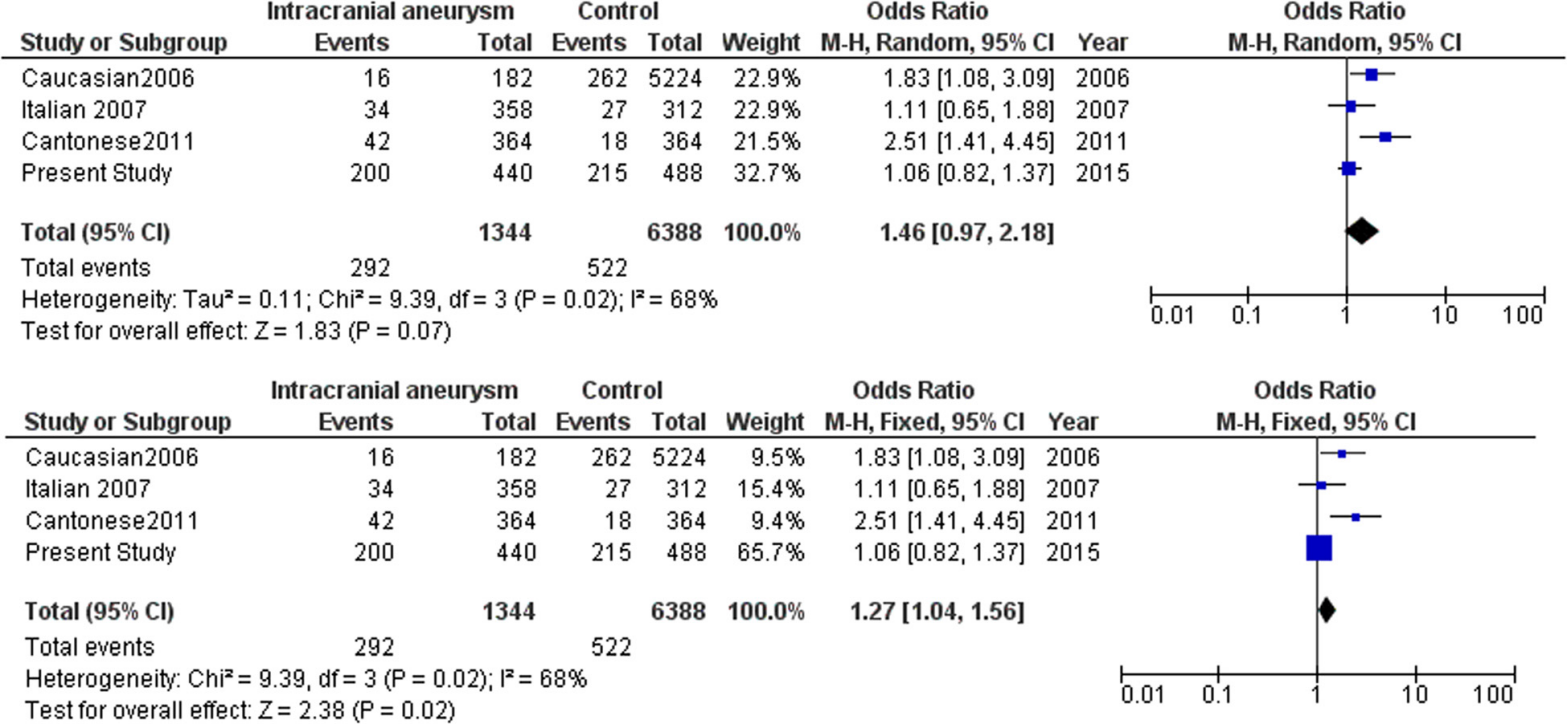
Meta-analysis of *IL6* rs1800796 (−572C/G) C versus G allele in patients with intracranial aneurysm versus normal control in C allele-associated population. Random and fixed effect models

**Fig. 5:**
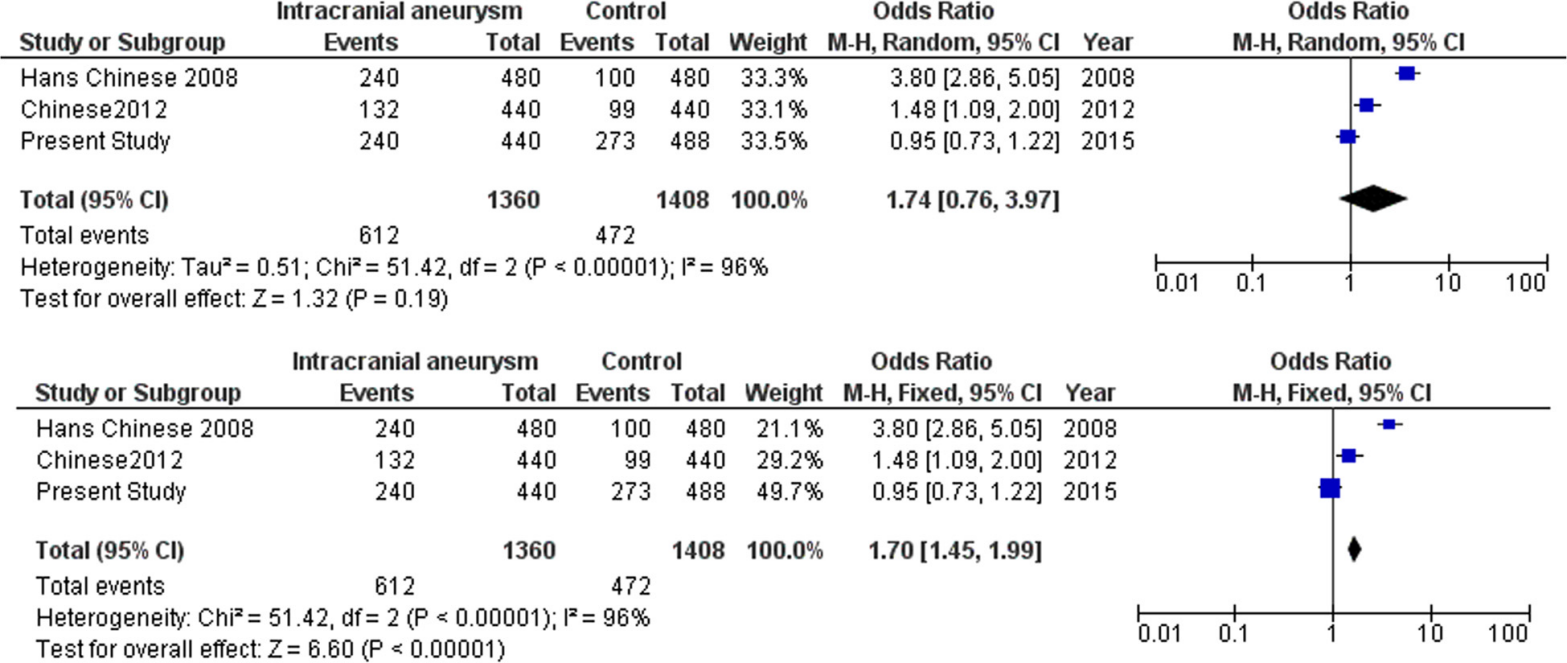
Meta-analysis of *IL6* rs1800796 (−572C/G) G versus C allele in patients with intracranial aneurysm versus normal control in C allele-associated population. Random and fixed effect models

**Fig. 6:**
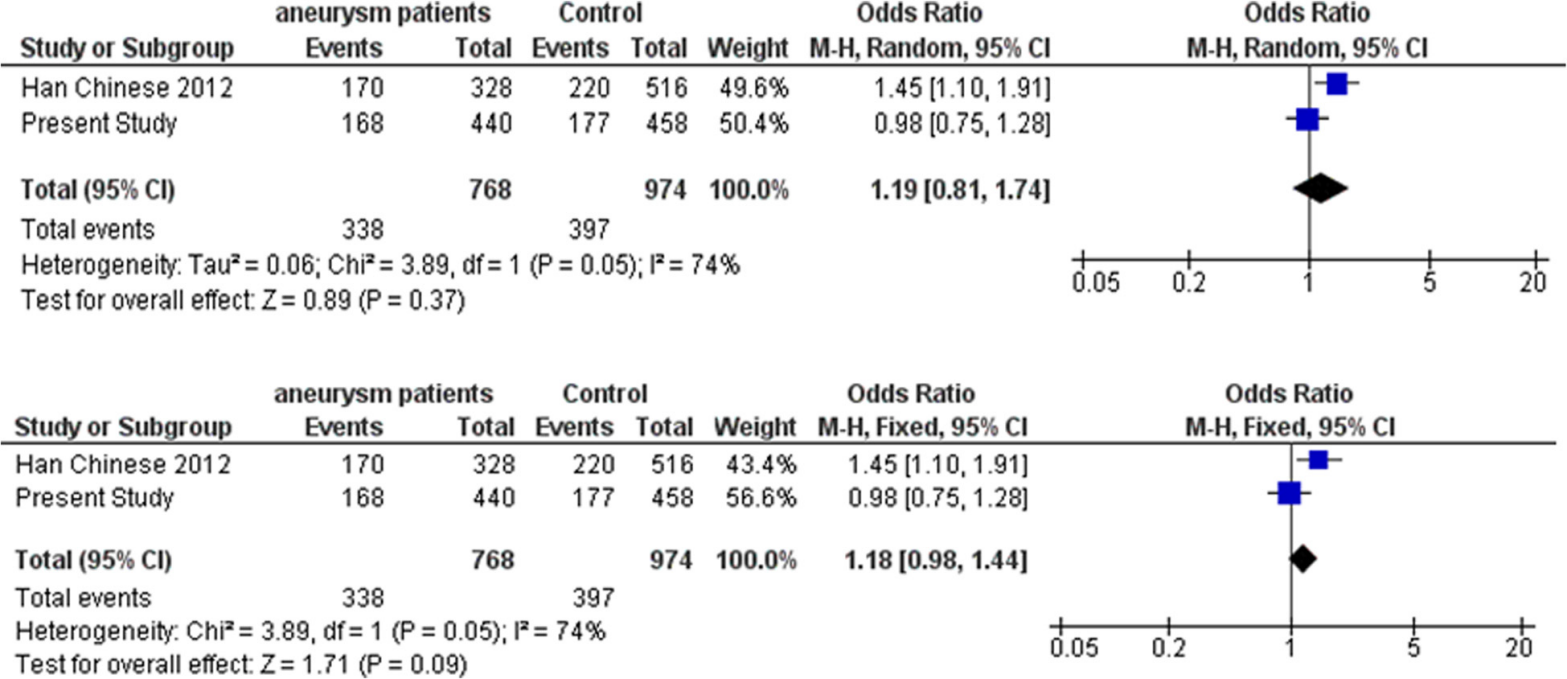
Meta-analysis of *IL12B* gene variant rs3212227 with intracranial aneurysm. Random and fixed effect models

**Fig. 7:**
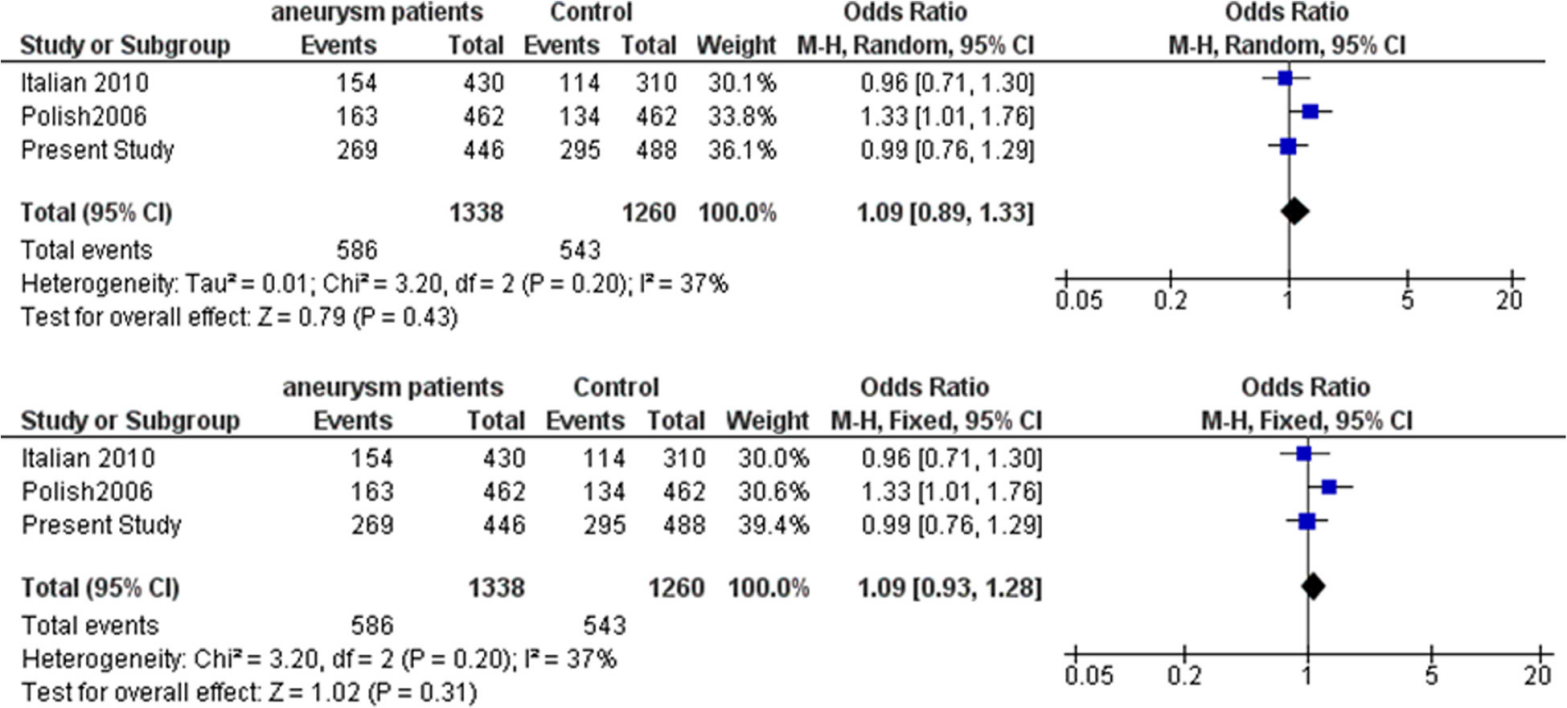
Meta-analysis of *IL1B* gene variant rs16944 with intracranial aneurysm. Random and fixed effect models

Meta-analysis with the SNPs in the cytokine genes was carried out with data from earlier studies and including the present study. Meta-analysis with the promoter polymorphism rs1800796 (−572C/G) of *IL6* indicated high heterogeneity between studies (*I*^2^ = 93 %) which accounts for the large variation in different studies shown by opposite alleles being associated in different world population. This prompted us to carry out meta-analysis using both random and fixed effect models. While comparing C versus G allele between IA patients and normal control, significant association was observed in fixed effect model (*Z* = 3.57, *p* = 0.0004) while no association was observed in random effect model (*Z* = 0.02, *p* = 0.98) (Fig. 3). Further stratification of studies into two groups was carried out based on the associated allele (group A: C versus G allele in IA patients versus control in C allele-associated population (Fig. 4) and group B: G versus C allele in IA patients versus control in C allele-associated population) (Fig. 5). This was specifically done as G allele was associated in Han Chinese ethnicity while C allele was found to be associated in Caucasian and Cantonese ethnicity. Association was observed with both C allele (*Z* = 2.38, *p* = 0.02) and G allele (*Z* = 6.60, p < 0.00001) with fixed effect model (Figs. 4 and 5). Meta-analysis with rs3212227 of *IL12B* and rs16944 of *IL1B* did not show any association in both fixed effect and random effect models (Figs. 6 and 7).

## Discussion

The present study explores the genetic liability to inflammatory response induced by genetic variability in cytokine genes and how this genetic variability through differential inflammatory response could result in the development of aneurysm. The study comprised of pro-inflammatory cytokines *IL1*, *IFNG*, *TNFA*, and *IL6*; anti-inflammatory cytokines *IL1RN*, *TGFB1*, and *IL10*; and pleiotropic cytokines *IL3*, *IL12B*, and *IL4* genes. We report a novel association with pro-inflammatory cytokines *TNFA* and *IFNG*. Interestingly, some of the reported association with the pro-inflammatory cytokines of *IL1* gene cluster and *IL6* were not found to be associated in the present study. None of the pleiotropic cytokines were associated with IA in the present study, but we did observe a novel association with anti-inflammatory cytokines *IL10.* Most of the studies till date were restricted to individual cytokines and mainly focused on *IL1B*, *IL6*, and *TNFA* which could not be replicated in different ethnicities. We could not find any significant association for subgroup analysis which suggests that the associated SNPs might have a direct effect on the development of IA and was independent of hypertension and gender. Limitation of the study was with large effect size, and this would involve increased sample size. Large effect sizes are uncommon in complex diseases and therefore multiple testing was not applied to the study. Increasing sample size would mean sampling genetically non-stratified sample which will further compromise the observation. This is also the reason why genetic studies are not replicable across ethnicities, and this conflict is evident in the meta-analysis due to genetic heterogeneity. Monitoring the cytokine levels of these associated cytokines might be of interest in IA pathogenesis as the immunogenetic background might suggest an individual’s response to a given immunological stress in the development and progression of aneurysm. The differential inflammatory response mediated by genetic variants in cytokines could be a primary event, while inflammatory response mediated by pre- or posttraumatic stress or comorbid factors could further augment the disease pathology.

Association with *TNFA* polymorphic variant in the present study is interesting as it is known to be a powerful pro-inflammatory cytokine. Expression studies indicate overexpression of *TNFA* to modulate susceptibility to IA [18]. While IA is known to be a multifactorial disease, similarly, the production and modulatory role of TNF is also multistimulatory. Therefore, how *TNFA* promotes pathogenesis of IA is indeed a challenge. *In-Silico* functional prediction suggests that rs361525 variant is involved in transcriptional regulation with FS score of 0.208. Functional studies with rs361525 show differential effect of transcriptional activation with *TNFA* rs361525-238A allele conferring increased transcriptional activation of the TNF promoter [19]. “A” allele of rs361525 has been reported to enhance the responsiveness of *TNFA* promoter by 2.2 to 2.8 times greater versus the “G” variant, when stimulated with pro-inflammatory stimuli [19]. Functional validation studies further suggests that “A” allele of rs361525 creates a functionally active thyroid hormone receptor (TR) binding site which resulted in TR-α-induced promoter overexpression by 1.8-fold. Earlier studies on *TNFA* provided evidence for rs1800629 (−308 G<A) with IA in Italian population, but this SNP was not found to be associated with our study population [20]. It has been reported that among *TNFA* promoter polymorphisms, rs361525 could modulate transcriptional regulation but not the rs1800629 [21]. Therefore, association with “A” allele of rs361525 in the present study could indicate overproduction of TNFα as a genetic event and thereby mediating development and rupture of IA in south Indian population. Risk factors that associate with intracranial aneurysm such as hemodynamic stress [22], hypertension [23], and smoking [24] are also known to induce the production of TNFα. Increased stimulation of TNFα and Fas-associated death domain protein, which are involved in inflammation and apoptosis, can have deleterious effect on arterial wall [18]. Thus, in the present study, the over representation of A allele which refers to increased transcriptional activation of TNF promoter may result in the development of aneurysm or aneurismal rupture, through its multistimulatory role induced by multifactorial nature of IA.

Genetic association of *TNFA* and *IFNG* may further exacerbate the pro-inflammatory role as *TNFA* is known to synergize with *IFNG* in inhibiting the proliferation of various cell types. The growth inhibitory activities of *IFNG* are more pronounced in endothelial cells. Polymorphisms in the promoter regions of *IFNG* have been reported to influence IFN-γ production. In the present study, we report higher representation of *IFNG* +3234TT genotype in aneurismal patients. Based on the functional studies of +3234C/T polymorphism, +3234C alleles has been reported to be associated with a higher level of IFN-γ than the +3234T alleles [25]. Higher frequency of homozygous +3234TT genotype could be indicative of lower levels of IFN-γ. The regulation of *IFNG* gene transcription is complex and several transcription factors have been found to interact with the proximal promoter constitutively [26] or by induction [27]. One of the most important functions of *IFNG* is macrophage activation function, which upregulates the expression of the major histocompatibility complexes (MHC)I [28] and MHCII [29], involved in antigen processing and presentation pathways. IFN-γ also mediates functions such as leukocyte attraction [28], maturation and differentiation, natural killer (NK) cell activity [30], and immunoglobulin (Ig) production and class switching in B cells [28].Therefore, reduced IFN-γ production would result in compromised resultant functions such as reduced leukocyte attraction or macrophage activation.

Pro-inflammatory cytokines are linked in a sequence resembling an electrical circuit in series, with TNF-α at the apex of the cascade inducing production of IL-1, and subsequently both TNF-α and IL-1 induce downstream cytokines such as IL-6 [31]. Polymorphisms in any of these genes are likely to alter the downstream functional events. Earlier studies did provide evidence with contradictions for involvement of *IL1* gene complex with IA. A study in Polish population has reported an association of *IL1B* rs16944 with IA, but this could not be replicated in Italian population [16, 17]. *IL1RN* VNTR polymorphism has been widely implicated in ischemic stroke [32] and coronary artery disease (CAD) [33]. In the present study, we failed to replicate these observations in IA patients from south India. Another pro-inflammatory cytokine *IL6* had been the point of intense study by several investigators. Morgan et al. (2006) first reported that *IL6* promoter polymorphism rs1800796 (−572C/G) and rs1800795 (−174G/C) are associated with IA in Caucasian population and 572C/174C haplotype having a greater risk [10]. Subsequent studies with these variants in Italian and Chinese population had shown conflicting results [11–14]. While rs1800796 and rs1800795 were not associated with IA in Italian population [11], it was a conflict of allelic associations in Chinese population. In Han Chinese [12] and west Chinese population [14], G allele proved to be a risk allele, whereas in Cantonese population, C allele turned out to be a risk allele for IA [13]. None of these allelic associations for *IL6* could be replicated in our study population for IA. Observations from some of these extensively investigated proinflammatory cytokines remained inconclusive; therefore, we carried out a meta-analysis with these SNPs by including the present study. Meta-analysis with rs3212227 of *IL12B* and rs16944 of *IL1B* did not show any association in both fixed effect and random effect models, while with *IL6* indicated tremendous heterogeneity between studies which could be accounted to large variation in different studies shown by opposite alleles being associated in different world population. Therefore, conflicting allelic association of *IL6* needs to be carefully interpreted in terms of its functional significance to environment and comorbid factors that can influence IL6 production and subsequently result in development of IA. These ethnic-specific genetic associations are important to understand the complex nature of the disease which might help in modeling the gene-environment interaction. All these studies indicate that the cascade of events in pro-inflammatory cytokine circuit is active, but sequence of events in the circuit tends to differ in different ethnicities in developing risk for IA.

None of the anti-inflammatory or pleiotropic cytokines were found to be associated at allelic or genotypic combinations in the present study with exception to *IL10*. In *IL10*, the heterozygous genotypes of the two tagged SNPs rs1800871 and rs1800872 were observed to be associated with IA. *IL10* is a potent anti-inflammatory and immunomodulatory cytokine, exerting major effects in the degree and quality of the immune response. It is a potent suppressor of pro-inflammatory cytokines such as *IL1*, *IL6*, *IL12*, *TNFA*, and *IFNG* and inhibits the costimulatory activity of macrophages for T cell and NK cell activation. It is monoallelically expressed in CD4+ T cells. Evidences suggest that the monoallelic expression pattern of *IL10* is not due to parental imprinting, allelic exclusion, or strong allelic bias, instead, due to stochastic regulation mechanism, in which the probability to initiate allelic transcription depends on the strength of TCR signaling and subsequent capacity to overcome restrictions imposed by chromatin hypoacetylation. Allelic expression data shows transcriptional independence between both alleles [34]. This could possibly help us understand why in an earlier study, *IL10* was not found to express in IA patients [18]. Monoallelic expression and stochastic regulation of *IL10* might help us understand the functional implication of heterozygous genotype with IAs, observed in the present study. The rs1800872 variant has been reported to be a low-producer allele of the *IL10* gene [35]. A recent study have reported that approximately one third of the genes have the same allele expressed more highly in all heterozygotes, suggesting that their regulation is predominantly influenced by cis elements in strong linkage disequilibrium with the assayed exonic SNP. The remaining two thirds of the genes have different alleles expressed more highly in different heterozygotes, suggesting that their expression differences are influenced by factors not in strong linkage disequilibrium with the assayed exonic SNP [36]. Interestingly, stochastic monoallelic expression of *IL10* has been reported which are transcriptionally independent [34]. Based on these observations, one can suggest that the pathologic modulation of *cis*- or the *trans*-acting factors on the heterozygous genotypes might result in the development of aneurysm.

## Conclusion

We conclude that genetic liability of anti-inflammatory *IL10* rs1800871 and rs1800872 variant in combination with pro-inflammatory *TNFA* rs361525 and *IFNG* rs2069718 gene variant may promote pathogenesis of IA. The differential inflammatory response mediated by genetic variants in cytokines could be a primary event, while inflammatory response mediated by stochastic events of pre- or post-traumatic stress or comorbid factors could further augment the disease pathology. It has been reported that locally secreted cytokines may tilt the regional balance of matrix metalloproteinases (MMP) activity in favor of vascular matrix degradation [37]. Therefore, chronic exposure to pro-inflammatory cytokines *TNFA* and *IFNG* and stochastic regulation of *IL10* and *TGFB* in response to pathologic environment may support development of IA. The implication and interaction of these genetic variants under a specific environmental background will help us identify the resultant phenotypic variation in the pathogenesis of intracranial aneurysm. Identifying genetic risk factors for inflammation might also help in understanding and addressing the post-traumatic complications following the aneurismal rupture.

## Additional file

Additional file 1:
**Supplementary information.** List of the selected SNPs and the genotyping method employed **(Table S1)**. List of studies included in meta-analysis **(Table S2)**. Comparison of the genotype and allele frequencies of cytokine gene variants between patients and control **(Table S3)**. In silico functional prediction of relevant SNPs using F-SNP score **(Table S4)**. Genetic association analysis with studied variants between male and female IA patients **(Figure S1)**. SNPs were plotted against –log(*p* value).
