# Erratum to: Pathogenesis of intracranial aneurysm is mediated by proinflammatory cytokine TNFA and IFNG and through stochastic regulation of IL10 and TGFB1 by comorbid factors

**DOI:** 10.1186/s12974-015-0383-8

**Published:** 2015-09-15

**Authors:** Sanish Sathyan, Linda V. Koshy, Lekshmy Srinivas, H. V. Easwer, S. Premkumar, Suresh Nair, R. N. Bhattacharya, Jacob P. Alapatt, Moinak Banerjee

**Affiliations:** Human Molecular Genetics Laboratory, Rajiv Gandhi Centre for Biotechnology, Thiruvananthapuram, 695014, Kerala India; Department of Neurosurgery, Sree Chitra Tirunal Institute for Medical Science and Technology, Thiruvananthapuram, Kerala India; Department of Neurosurgery, Calicut Medical College, Calicut, Kerala India

## Erratum

Following publication of [1] it came to the authors’ attention that Lekshmy Srinivas’ name was displayed incorrectly. The correct spelling of their name is included in this erratum.
