# Supplementary material for: Pathogenesis of intracranial aneurysm is mediated by proinflammatory cytokine TNFA and IFNG and through stochastic regulation of IL10 and TGFB1 by comorbid factors
Source: J Neuroinflammation. 2015 Jul 22;12:135. doi: 10.1186/s12974-015-0354-0 (PMC4510902; doi:10.1186/s12974-015-0354-0)

**Supplementary Table-1:** **List of cytokine candidate genes and their selected polymorphisms with their predicted functional effect.**

| **Gene** | **Locus** | **SNP** | **rs id** | **Alleles** | **Method** | **Location** | **Predicted Functional Effect** |
| --- | --- | --- | --- | --- | --- | --- | --- |
| **IL1A** | 2q13 | -889G>A | rs1800587 | G/A | TaqMan allelic | Promoter | TFBS,Splicing(ESE or ESS) |
| **IL1B** | 2q13 | +3954 C>T, Phe105Phe | rs1143634 | C/T | PCR-RFLP | Exon 4 | sSNP, Splicing(ESE or ESS) |
|  |  | –31T>C | rs1143627 | C/T | PCR-RFLP | Promoter | TFBS |
|  |  | –511C>T | rs16944 | T/C | PCR-RFLP | Promoter | TFBS |
| **IL1RN** | 2q13 | (86)_n_ VNTR | - | 1/ 2 | PCR FLP | Intron 4 | Regulatory protein-binding sites |
| **IL4** | 5q31.1 | -590C>T | rs2243250 | C/T | PCR-RFLP | Promoter | TFBS |
|  |  | -33C>T | rs2070874 | C/T | TaqMan | Promoter | TFBS |
| ***IL6*** | 7p21 | –597G>A | rs1800797 | G/A | PCR-RFLP | Promoter | TFBS |
|  |  | –572G>C | rs1800796 | G/C | PCR-RFLP | Promoter | TFBS |
|  |  | –174G>C | rs1800795 | G/C | PCR-RFLP | Promoter | TFBS |
| **IL10** | 1q31 | –592A>C | rs1800872 | T/G | PCR-RFLP | Promoter | TFBS |
|  |  | –819C>T | rs1800871 | G/A | KASPar assay | Promoter | TFBS |
|  |  | –1082G>A | rs1800896 | T/C | KASPar assay | Promoter | TFBS |
| **IL3** | 5q31.1 | rs31400T>C | rs31400 | T/C | TaqMan | Promoter | Promoter/regulatory region |
|  |  | rs31480C>T | rs31480 | C/T | TaqMan | Promoter | TFBS |
|  |  | rs40401C>T | rs40401 | C/T | TaqMan | Exon 1 | nsSNP, benign |
| ***TNFA*** | 6p21.3 | –308G>A | rs1800629 | G/A | KASPar assay | Promoter | TFBS |
|  |  | –238G>A | rs361525 | G/A | KASPar assay | Promoter | TFBS |
| **IFNG** | 12q24.1 | +3232A>G | rs2069718 | A/G | TaqMan | Intron 3 | -- |
|  |  | +874A>T | rs2430561 | A/T | KASPar assay | Intron 1 | NFKB binding site |
| **TGFB1** | 19q13.2 | +915G>C, Arg25Pro | rs1800471 | G/C | KASPar assay | Exon 1 | nsSNP, benign |
|  |  | +869A>G, Leu10Pro | rs1800470 | A/G | TaqMan allelic | Exon 1 | nsSNP |
|  |  | –509C>T | rs1800469 | C/T | KASPar assay | Promoter | TFBS |

**Supplementary Table 2:** List of studies included in meta-analysis

| **GENE** | **SNP** | **Author** | **Population** | **Year** |
| --- | --- | --- | --- | --- |
| IL6 | rs1800796 -572G/C | Morgan et al | Caucasian | 2006 |
|  |  | Fontanella et al | Italian | 2007 |
|  |  | Sun et al | Chinese Han | 2008 |
|  |  | Zhang et al | Cantonese | 2011 |
|  |  | Liu et al | Chinese | 2012 |
|  |  | Present study | Indian | 2014 |
| IL12B | rs3212227 | Li et al | Han Chinese | 2012 |
|  |  | Present study | Indian | 2014 |
| IL1B | rs16944 | Slowik et al | Polish | 2006 |
|  |  | Fontanella et al | Italian | 2010 |
|  |  | Present study | Indian | 2014 |

**Supplementary Table 3:** Comparison of the genotype and allele frequencies of gene variants in between patients and control

| **IFNG** |  | **AA** | **AG** | **GG** | **p-value** | **A** | **G** | **OR(95%CI)** | **p-value** |
| --- | --- | --- | --- | --- | --- | --- | --- | --- | --- |
|  | **Cases** | 79 | 103 | 39 |  | 261 | 181 |  |  |
| **rs2069718** |  | 0.36 | 0.47 | 0.18 | **0.020** | 0.59 | 0.41 | **1.203(0.927 to 1.560)** | **0.184** |
|  | **Controls** | 61 | 143 | 39 |  | 265 | 221 |  |  |
|  |  | 0.25 | 0.59 | 0.16 |  | 0.55 | 0.45 |  |  |
| **IFNG** |  | **AA** | **AT** | **TT** |  | **A** | **T** |  |  |
|  | **Cases** | 78 | 101 | 31 |  | 257 | 163 |  |  |
| **rs2430561** |  | 0.37 | 0.48 | 0.15 | 0.307 | 0.61 | 0.39 | **0.838(0.639 to 1.099)** | **0.213** |
|  | **Controls** | 107 | 102 | 33 |  | 316 | 168 |  |  |
|  |  | 0.44 | 0.42 | 0.14 |  | 0.65 | 0.35 |  |  |
| **TNF** |  | **AA** | **AG** | **GG** |  | **A** | **G** |  |  |
|  | **Cases** | 0 | 41 | 185 |  | 41 | 411 |  |  |
| **rs1800629** |  | 0.00 | 0.18 | 0.82 | 0.465 | 0.09 | 0.91 | **0.818(0.533 to 1.258)** | **0.213** |
|  | **Controls** | 1 | 51 | 192 |  | 53 | 435 |  |  |
|  |  | 0.00 | 0.21 | 0.79 |  | 0.11 | 0.89 |  |  |
|  |  | **GG** | **AG** | **AA** |  | **G** | **A** |  |  |
|  | **Cases** | 171 | 42 | 2 |  | 384 | 46 |  |  |
| **rs361525** |  | 0.80 | 0.20 | 0.01 | **0.019** | 0.89 | 0.11 | **1.888(1.163 to 3.064)** | **0.010** |
|  | **Controls** | 216 | 25 | 2 |  | 457 | 29 |  |  |
|  |  | 0.89 | 0.10 | 0.01 |  | 0.94 | 0.06 |  |  |
| **IL3** |  | **CC** | **CT** | **TT** |  | **C** | **T** |  |  |
|  | **Cases** | 108 | 92 | 22 |  | 308 | 136 |  |  |
| **rs31480** |  | 0.49 | 0.41 | 0.10 | 0.89 | 0.69 | 0.31 | **0.944(0.713 to 1.250)** | **0.720** |
|  | **Controls** | 121 | 101 | 21 |  | 343 | 143 |  |  |
|  |  | 0.50 | 0.42 | 0.09 |  | 0.71 | 0.29 |  |  |
|  |  | **CC** | **CT** | **TT** |  | **C** | **T** |  |  |
|  | **Cases** | 19 | 112 | 84 |  | 150 | 280 |  |  |
| **rs31400** |  | 0.09 | 0.52 | 0.39 | 0.055 | 0.35 | 0.65 | 0.8435(0.644 to 1.105) | 0.217 |
|  | **Controls** | 39 | 110 | 93 |  | 188 | 296 |  |  |
|  |  | 0.16 | 0.45 | 0.38 |  | 0.39 | 0.61 |  |  |
|  |  | **CC** | **CT** | **TT** |  | **C** | **T** |  |  |
|  | **Cases** | 99 | 83 | 23 |  | 281 | 129 |  |  |
| **rs40401** |  | 0.48 | 0.40 | 0.11 | 0.786 | 0.69 | 0.31 | 0.9563(0.718 to 1.273) | 0.770 |
|  | **Controls** | 114 | 100 | 22 |  | 328 | 144 |  |  |
|  |  | 0.48 | 0.42 | 0.09 |  | 0.69 | 0.31 |  |  |
| **TGFB** |  | **GG** | **GC** | **CC** |  | **G** | **C** |  |  |
|  | **Cases** | 52 | 79 | 36 |  | 183 | 151 |  |  |
| **TGFBrs1800470** |  | 0.31 | 0.47 | 0.22 | 0.864 | 0.55 | 0.45 | 1.018(0.769-1.348) | 0.943 |
|  | **Controls** | 71 | 121 | 50 |  | 263 | 221 |  |  |
|  |  | 0.29 | 0.50 | 0.21 |  | 0.54 | 0.46 |  |  |
|  |  | **GG** | **GC** | **CC** |  | **G** | **C** |  |  |
|  | **Cases** | 186 | 35 | 1 |  | 407 | 37 |  |  |
| **TGFBrs1800471** |  | 0.84 | 0.16 | 0.00 | 0.268 | 0.92 | 0.08 | 0.695(0.419 to 1.151) | 0.161 |
|  | **Controls** | 215 | 29 | 0 |  | 459 | 29 |  |  |
|  |  | 0.88 | 0.12 | 0.00 |  | 0.94 | 0.06 |  |  |
|  |  | **CC** | **CT** | **TT** |  | **C** | **T** |  |  |
|  | **Cases** | 88 | 102 | 32 |  | 278 | 166 |  |  |
| **TGFBrs1800469** |  | 0.40 | 0.46 | 0.14 | 0.466 | 0.63 | 0.37 | 0.8864(0.680 to 1.155) | 0.381 |
|  | **Controls** | 92 | 104 | 45 |  | 288 | 194 |  |  |
|  |  | 0.38 | 0.43 | 0.19 |  | 0.60 | 0.40 |  |  |
| **IL6** |  | **GG** | **GC** | **CC** |  | **G** | **C** |  |  |
|  | **Cases** | 144 | 63 | 8 |  | 351 | 79 |  |  |
| **IL6rs1800795** |  | 0.67 | 0.29 | 0.04 | 0.625 | 0.82 | 0.18 | 0.8517(0.613 to 1.182) | 0.360 |
|  | **Controls** | 153 | 80 | 11 |  | 386 | 102 |  |  |
|  |  | 0.63 | 0.33 | 0.05 |  | 0.79 | 0.21 |  |  |
|  |  | **GG** | **GC** | **CC** |  | **G** | **C** |  |  |
|  | **Cases** | 57 | 126 | 37 |  | 240 | 200 |  |  |
| **IL6rs1800796** |  | 0.26 | 0.57 | 0.17 | 0.050 | 0.55 | 0.45 | 0.945(0.729 to 1.225) | 0.691 |
|  | **Controls** | 81 | 111 | 52 |  | 273 | 215 |  |  |
|  |  | 0.33 | 0.45 | 0.21 |  | 0.56 | 0.44 |  |  |
| **IL4** |  | **CC** | **CT** | **TT** |  | **C** | **T** |  |  |
|  | **Cases** | 166 | 62 | 3 |  | 394 | 68 |  |  |
| **IL4rs2070874** |  | 0.72 | 0.27 | 0.01 | 0.645 | 0.85 | 0.15 | 0.907(0.637 to 1.292) | 0.652 |
|  | **Controls** | 172 | 66 | 6 |  | 410 | 78 |  |  |
|  |  | 0.70 | 0.27 | 0.02 |  | 0.84 | 0.16 |  |  |
| **IL12B** |  | **AA** | **AC** | **CC** |  | **A** | **C** |  |  |
|  | **Cases** | 69 | 109 | 44 |  | 247 | 197 |  |  |
| **IL12Brs2853694** |  | 0.31 | 0.49 | 0.20 | 0.175 | 0.56 | 0.44 | 0.8534(0.653 to 1.114) | 0.248 |
|  | **Controls** | 86 | 91 | 44 |  | 263 | 179 |  |  |
|  |  | 0.39 | 0.41 | 0.20 |  | 0.60 | 0.40 |  |  |
|  |  | **AA** | **AC** | **CC** |  | **A** | **C** |  |  |
|  | **Cases** | 88 | 96 | 36 |  | 272 | 168 |  |  |
| **IL12Brs3212227** |  | 0.40 | 0.44 | 0.16 | 0.358 | 0.62 | 0.38 | 0.980(0.749 to 1.283) | 0.891 |
|  | **Controls** | 83 | 115 | 31 |  | 281 | 177 |  |  |
|  |  | 0.36 | 0.50 | 0.14 |  | 0.61 | 0.39 |  |  |
| **IL1A** |  | **GG** | **GA** | **AA** |  | **G** | **G** |  |  |
|  | **Cases** | 109 | 83 | 27 |  | 301 | 137 |  |  |
| **IL1Ars1800587** |  | 0.50 | 0.38 | 0.12 | 0.096 | 0.69 | 0.31 | 0.907(0.684 to 1.201) | 0.518 |
|  | **Controls** | 118 | 108 | 17 |  | 344 | 142 |  |  |
|  |  | 0.49 | 0.44 | 0.07 |  | 0.71 | 0.29 |  |  |
| **IL1RN** |  | **11** | **12** | **22** |  | **1** | **2** |  |  |
|  | **Cases** | 107 | 80 | 16 |  | 294 | 112 |  |  |
| **IL1RN-VNTR** |  | 0.53 | 0.39 | 0.08 | 0.476 | 0.72 | 0.28 | 0.995(0.741 to 1.337) | 1 |
|  | **Controls** | 133 | 84 | 25 |  | 350 | 134 |  |  |
|  |  | 0.55 | 0.35 | 0.10 |  | 0.72 | 0.28 |  |  |
| **IL1B** |  | **CC** | **CT** | **TT** |  | **C** | **T** |  |  |
|  | **Cases** | 79 | 82 | 33 |  | 240 | 148 |  |  |
| **IL1Brs1143627** |  | 0.41 | 0.42 | 0.17 | 0.520 | 0.62 | 0.38 | 1.041(0.791 to 1.369) | 0.780 |
|  | **Controls** | 90 | 116 | 37 |  | 296 | 190 |  |  |
|  |  | 0.37 | 0.48 | 0.15 |  | 0.61 | 0.39 |  |  |
|  |  | **AA** | **AG** | **GG** |  | **C** | **T** |  |  |
|  | **Cases** | 84 | 101 | 38 |  | 269 | 177 |  |  |
| **IL1Brs16944** |  | 0.38 | 0.45 | 0.17 | 0.912 | 0.60 | 0.40 | 0.994(0.764 to 1.293) | 1 |
|  | **Controls** | 90 | 115 | 39 |  | 295 | 193 |  |  |
|  |  | 0.37 | 0.47 | 0.16 |  | 0.60 | 0.40 |  |  |
| **IL10** |  | **AA** | **AG** | **GG** |  | **A** | **G** |  |  |
|  | **Cases** | 146 | 71 | 8 |  | 363 | 87 |  |  |
| **IL10rs1800896** |  | 0.65 | 0.32 | 0.04 | 0.402 | 0.81 | 0.19 | 0.832(0.606 to 1.141) | 0.262 |
|  | **Controls** | 151 | 80 | 15 |  | 382 | 110 |  |  |
|  |  | 0.61 | 0.33 | 0.06 |  | 0.78 | 0.22 |  |  |
|  |  | **CC** | **CT** | **TT** |  | **C** | **T** |  |  |
|  | **Cases** | 48 | 142 | 40 |  | 238 | 222 |  |  |
| **IL10rs1800871** |  | 0.21 | 0.62 | 0.17 | 0.016 | 0.52 | 0.48 | 1.081(0.837 to 1.396) | 0.558 |
|  | **Controls** | 61 | 118 | 62 |  | 240 | 242 |  |  |
|  |  | 0.25 | 0.49 | 0.26 |  | 0.50 | 0.50 |  |  |
|  |  | **AA** | **AC** | **CC** |  | **A** | **C** |  |  |
|  | **Cases** | 39 | 143 | 48 |  | 221 | 239 |  |  |
| **IL10rs1800872** |  | 0.17 | 0.62 | 0.21 | 0.005 | 0.48 | 0.52 | 0.9555(0.740 to 1.233) | 0.745 |
|  | **Controls** | 62 | 116 | 66 |  | 240 | 248 |  |  |
|  |  | 0.25 | 0.48 | 0.27 |  | 0.49 | 0.51 |  |  |

**Supplementary Table 4.** In-silico functional prediction of relevant SNPs in this study using F-SNP program

|  | Functional Category | Prediction Tool | Prediction Result | FS  score |
| --- | --- | --- | --- | --- |
| TNFArs361525 | transcriptional_regulation | TFSearch | changed | 0.208 |
|  |  | Consite | changed |  |
|  |  | GoldenPath | not exist |  |
| TNFArs1800629 | transcriptional_regulation | TFSearch | changed | 0.208 |
|  |  | Consite | changed |  |
|  |  | GoldenPath | not exist |  |
| IL3rs31480 | transcriptional_regulation | TFSearch | changed | 0.242 |
|  |  | GoldenPath | exist |  |
| TGFB1rs1800469 | transcriptional_regulation | TFSearch | changed | 0.208 |
|  |  | Consite | changed |  |
|  |  | GoldenPath | not exist |  |
| IL6rs1800795 | transcriptional_regulation | Ensembl-NS | frameshift_coding | 0.398 |
|  |  | GoldenPath | not exist |  |
|  |  | Ensembl-TR | regulatory_region |  |
| IL6rs1800796 | transcriptional_regulation | TFSearch | changed | 0.208 |
|  |  | Consite | changed |  |
|  |  | GoldenPath | not exist |  |
| IL12Brs2853694 | transcriptional_regulation | TFSearch | changed | 0.242 |
|  |  | GoldenPath | exist |  |
| IL12Brs3212227 | transcriptional_regulation | TFSearch | changed | 0.242 |
|  |  | GoldenPath | exist |  |
| IL1Ars1800587 | transcriptional_regulation | TFSearch | changed | 0.208 |
|  |  | GoldenPath | not exist |  |
| IL1Brs1143627 | transcriptional_regulation | Ensembl-NS | frameshift_coding | 0.5 |
|  |  | TFSearch | changed |  |
|  |  | GoldenPath | exist |  |
|  |  | Ensembl-TR | regulatory_region |  |
| IL1Brs16944 | transcriptional_regulation | TFSearch | not changed | 0.065 |
|  |  |  |  |  |
|  |  | GoldenPath | exist |  |
| IL10rs1800896 | transcriptional_regulation | GoldenPath | exist | 0.101 |
| IL10rs1800871 | transcriptional_regulation | GoldenPath | exist | 0.101 |

FS Score; Functional significance score

F-SNP: <http://compbio.cs.queensu.ca/F-SNP/>

**Supplementary Figure 1:** Genetic association analysis with studied variants between male and female IA patients. SNPs were plotted against –log(p-value)


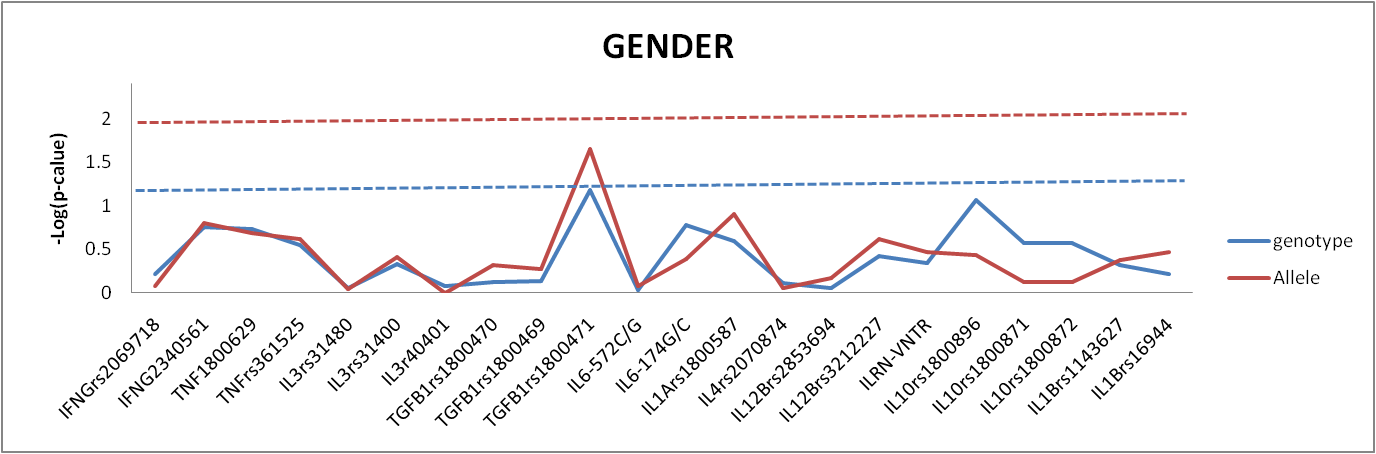

Supplement: Additional file 1: — Supplementary information. List of the selected SNPs and the genotyping method employed (Table S1). List of studies included in meta-analysis (Table S2). Comparison of the genotype and allele frequencies of cytokine gene variants between patients and control (Table S3). In silico functional prediction of relevant SNPs using F-SNP score (Table S4). Genetic association analysis with studied variants between male and female IA patients (Figure S1). SNPs were plotted against –log(p value). [file 12974_2015_354_MOESM1_ESM.docx]
